# A survey of the use of seclusion and physical restraint at school and at home for children under the care of the NHS Lanarkshire CAMHS – learning disability team

**DOI:** 10.1192/bjo.2021.592

**Published:** 2021-06-18

**Authors:** Sukhmeet Singh, Judith McKie

**Affiliations:** NHS Lanarkshire

## Abstract

**Aims:**

To attempt to quantify the use of seclusion and restraint for young people managed by the NHS Lanarkshire CAMHS Learning Disability team.

**Background:**

There has been increasing interest in the use of seclusion and restraint in children with learning disabilities, reflected by various news reports in the UK and USA. A survey of parents conducted by the Challenging Behaviour Foundation (2019) found that 35% of disabled children (n = 204) had been regularly physically restrained and a further 21% had been regularly secluded. The use of restrictive practice in children is contradictory to the UN Convention on the Rights of the Child as well as the conclusion of a recent report conducted by the Scottish Children's Commissioner titled “No Safe Place” which called on schools to stop using restraint and seclusion until national guidelines and standards were in place. No data were submitted to the Children's Commissioner from Lanarkshire. Anecdotally there was an impression in the team that restrictive practices were widely used at school and home.

**Method:**

The NHS Lanarkshire CAMHS-LD team is a small team caring for children aged 5–18 years with moderate to severe/profound learning disability with mental disorder and/or severe challenging behaviour. The methodology for this retrospective audit relied on reviewing patient case notes and speaking with involved clinicians. We discussed each individual patient on their respective caseload as to whether the child had been restrained or secluded at home or at school.

**Result:**

All 108 children from the caseload were included in the audit of whom 52.8% had been either restrained or secluded. 24.1% of children were both restrained and secluded at school, while 15.8% were restrained and secluded at home. These patients were complex. 86.1% had Autistic Spectrum Disorder and 55.6% had another comorbidity, such as ADHD.

**Conclusion:**

The figures are broadly similar to those in the Challenging Behaviour Foundation report. The team knew all of the individual patients very well and review them across a variety of settings such as school and home with instances of seclusion and restraint being directly witnessed by clinicians. Nevertheless, there is the issue of recall bias. These findings will be shared with NHS Lanarkshire management for further discussion and dissemination. .

